# Transcatheter closure of ruptured right-coronary aortic sinus fistula to right ventricle

**DOI:** 10.4103/0974-2069.74052

**Published:** 2010

**Authors:** Asaad Khoury, Ihab Khatib, Majdi Halabi, Avraham Lorber

**Affiliations:** 1 Department of Pediatric Cardiology, Rambam Health Care Campus, Technion-Institute of Technology, Haifa, Israel; 2 Department of Cardiology, Rambam Health Care Campus, Technion-Institute of Technology, Haifa, Israel; 3 The Ruth and Bruce Rappaport Faculty of Medicine, Technion-Institute of Technology, Haifa, Israel

**Keywords:** Fistula, transcatheter, valsalva sinus

## Abstract

A 22-year-old man was referred for evaluation of exertional fatigue. On examination, there were no overt signs of congestive heart failure. Transthoracic and transesophageal echocardiography revealed rupture of the right coronary aortic sinus of Valsalva into the right ventricle. It was successfully closed with a 12 × 10 Amplatzer duct occluder.

## INTRODUCTION

A ruptured aneurysmatic fistula of the coronary sinus has been considered a very rare cardiac lesion. The treatment of choice of such fistulae is a surgical repair, but with the recent advances in the transcatheter treatment of the left to right shunts by occluder devices, the non-surgical treatment of coronary sinus fistulae is increasing.

We present a case of transcatheter treatment of right coronary aortic sinus of Valsalva to the right ventricle (RCAS-RV) fistula using Amplatzer duct occluder (ADO).

## CASE REPORT

A 22-year-old man was referred for evaluation of exertional fatigue. He was previously diagnosed with a ventricular septal defect (VSD). He was not on any medical therapy. On examination, there were no overt signs of congestive heart failure. A systolic precordial thrill was palpable and a harsh 5/6 continuous murmur was audible. The pulse was high volume with blood pressure of 135/65 mmHg. Electrocardiogram (ECG) demonstrated sinus rhythm and incomplete right bundle branch block.

Transthoracic echocardiography (TTE) revealed normal situs and connections with marked left ventricular (LV) enlargement; LV end diastolic/end systolic dimensions (LVEDd/LVESd) were 62/33 mm. No hypertrophy and no regional wall motion anomalies were noted and there was hyperactive LV function with LV fractional shortening of 47%. A sizable fistula with a large flow was detected shunting from RCAS-RV. There was no VSD or aortic valve regurgitation (AR).

**Figure 1:**
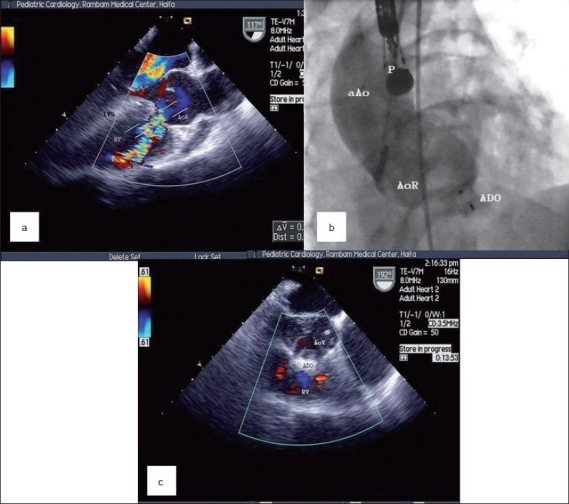
(a) TEE with color Doppler showing fistula opeining into the right ventricle (RV); (b) Full deployment of the 12 × 10 mm Amplatzer Duct Occluder (ADO) in the fistula after release; (c) TEE of the ADO *in situ* occluding the shunt (IVS, intraventricular septum; AoR, aortic root; F, fistula; aAo, ascending aorta; P, probe of TEE)

**Figure 2:**
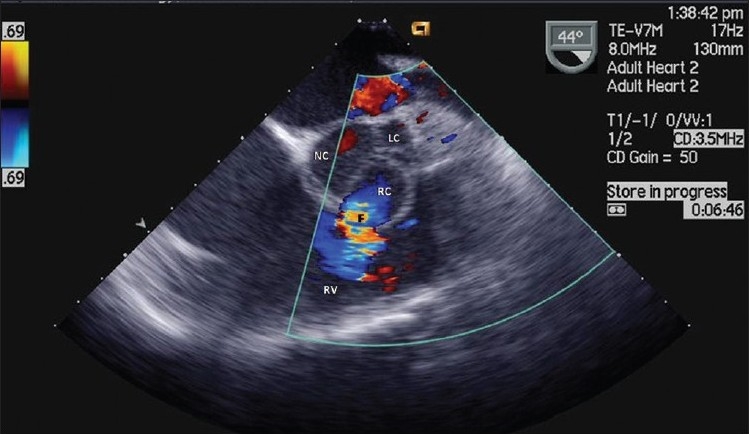
TEE view of right-coronary (RC) sinus fistula (F) into right ventricle (RV); left coronary (LC) and non-coronary (NC) cusps

The patient was scheduled for cardiac catheterization under transesophageal echo (TEE) guidance. The procedure was performed under general anesthesia with endotracheal intubation. The TEE interrogation disclosed an 8-mm fistula confirming the TTE findings [Figures 1a and 2]. A hemodynamic evaluation was performed with a normal pulmonary artery (PA) pressure 30/4 (15) mmHg and a QP/QS ratio of 3:1. The right anterior oblique projection aortogram illustrated the RCAS-RV irregular fistula which measured 8 mm in the aortic orifice and 9 mm in the mid portion.

**Figure 3:**
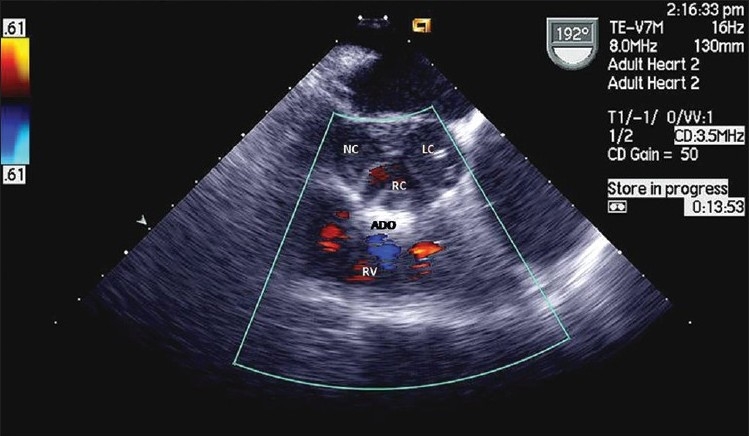
TEE view of ADO *in situ*; left coronary (LC), right coronary (RC) and non-coronary (NC) cusps

An arterio-venous loop was established by retrograde crossing of the fistula using a 0.025” Terumo guidewire (Terumo Medical Corporation, Somerset, NJ, USA) from the aorta to the RV followed by a multipurpose (MP) 5F catheter (Cordis, Miami, FL, USA). At this stage, a noodle wire (NW) (AGA Medical Corporation, Golden Valley, MN, USA) was introduced and was snared out from the pulmonary artery (PA) via the inferior vena cava (IVC) through the femoral vein using a 15-mm pfm snare (Produkte fur die Medizin AG. Cologne, Germany). An AGA 7F delivery sheath (AGADS) was introduced through the femoral vein to join the MP catheter in the IVC. The assembly of the MP with the AGADS was gently pushed over the NW to the level of the aortic arch. A 12 × 10 mm Amplatzer Duct Occluder (ADO) (AGA Medical Corporation, Golden Valley, MN, USA) was loaded into the AGADS with careful deairing of the system and the whole system was withdrawn to the ascending aorta. The ADO was partially deployed in the ascending aorta and the entire system was withdrawn to allow engagement of the 12-mm ADO disk with the aortic aspect of the RCAS-RV fistula. At that stage, the AGADS was further withdrawn to allow full deployment of the ADO. Following TEE evaluation of adequate ADO position and exclusion of AR, the ADO was released from the delivery system with TEE and aortogram confirming the final result [Figures 1b and 3]. A trivial color-flow Doppler residual shunt was noted on TEE [Figure 1c] and ascending aortography, immediately after ADO release, but it vanished on an outpatient evaluation 10 days following the procedure. No peri-procedural complications were noted. A very good LV function was maintained with LVEDd/LVESd 49/30 mm and marked reduction of LV size. Treatment with acetylsalicylic acid 100 mg daily was initiated after the procedure for 6 months.

## DISCUSSION

For patients with untreated aortic sinus Valsalva aneurysm rupture, the mean survival time after diagnosis has been estimated at 3.9 years.[1] The tissue at the aortic aspect of the fistula is frequently friable and difficult to manage, and surgical repair through the transaortic route may cause sinus of Valsalva distortion and aortic regurgitation[2] with survival rate close to 60% at 10 years during postoperative follow up.[3,4]

A few cases have been documented worldwide describing transcatheter closure of sinus of Valsalva fistulae. The first case was described in 1994 by Cullen *et al*. in a patient with recurrent sinus of Valasalva rupture after prior surgical repair using the Rashkind umbrella.[5] Fedson *et al*. had reported in 2003 the first case of the use of the ADO device to repair ruptured sinus of Valsalva aneurysm.[6] Since then, there have been a growing number of reports of nonsurgical closure using a duct occluder.[7–10]

Majority (77%) of the aneurysms of sinus of Valsalva arise from the right coronary sinus. The RV is the most common chamber of rupture (58%).[11] The noncoronary sinus is affected in 28% of patients.[12] About 20% of the noncoronary aneurysms rupture into RV, with only six cases being reported in two decades from a large center.[11,13] No comparative data with a long-term follow up are available for surgical repair and transcatheter closure of the different types of fistulae.

The technique of transcatheter closure of the RCAS-RV fistula presented here is proposed as an attractive alternative to surgery. The TEE or intravascular echo are mandatory modalities for adequate device deployment and release, avoiding complications such as inappropriate device position, device embolization, marked residual shunt and aortic valve regurgitation.

The option of device retrieval and replacement by a more suitable one increases the safety and the efficacy of this percutaneous approach. A failure of the percutaneous approach necessitates surgical intervention for closure of the fistula. Long term comparative data with surgical repair are necessary before recommending transcatheter closure as the procedure of choice for these individuals.
